# Clinical Characteristics and Risk Factors of an Outbreak with Scrub Typhus in Previously Unrecognized Areas, Jiangsu Province, China 2013

**DOI:** 10.1371/journal.pone.0125999

**Published:** 2015-05-08

**Authors:** Jianli Hu, Zhongming Tan, Dafei Ren, Xiang Zhang, Yilin He, Changjun Bao, Dapeng Liu, Qianhua Yi, Weijuan Qian, Jun Yin, Zhen Xu, Chunxia Yu, Shenjiao Wang, Bin Wu, Haiyu Yang, Ming Yue, Yun Zhang, Wendong Liu, Yefei Zhu, Minghao Zhou, Fenyang Tang

**Affiliations:** 1 Jiangsu Province Center for Disease Control and Prevention, Nanjing, China; 2 Tongren Municipal Center for Disease Control and Prevention, Tongren, China; 3 Taizhou Municipal Center for Disease Control and Prevention, Taizhou, China; 4 Jingjiang County Center for Disease Control and Prevention, Jingjiang, China; 5 The First Affiliated Hospital of Nanjing Medical University, Nanjing, China; 6 Huadong Research Institute for Medicine and Biotechnics, Nanjing, China; University of Texas Medical Branch, UNITED STATES

## Abstract

Scrub typhus, caused by *Orientia tsutsugamushi*, has emerged recently in Jingjiang City, China where the disease had not been known to exist. We analyzed epidemiological data, clinical characteristics and risk factors of scrub typhus outbreak in Jingjiang City, 2013. The 271 clinically diagnosed patients were predominantly farmers 50 to 69 years old and the peak of onset was early to mid-November. For the 187 laboratory-confirmed cases, the major clinical manifestations of the patients were fever (100%), eschar (88.2%), rash (87.7%), chills (87.7%), and headache (66.8%). A community-based case-control study was carried out to investigate the risk factors of the scrub typhus outbreak. Bundling or moving waste straw (OR=9.0, 95%CI 4.6-17.8) and living at the edge of village (OR=0.6, 95%CI 0.4-0.9) posed the highest risks through single- and multi-variable conditional logistic regression. Phylogenetic analysis of the 56-kDa TSA gene showed that the new cluster (GB-C2) and the previously reported cluster (GB-C1) of *O*. *tsutsugamushi* were associated with this outbreak. These findings are useful for the establishment of a detailed control strategy for scrub typhus infection in previously unrecognized areas of Jiangsu Province, China.

## Introduction

Scrub typhus is an acute, febrile and potentially fatal disease, caused by infection with the obligate intracellular bacterium *Orientia tsutsugamushi* (formerly *Rickettsia tsutsugamushi*), following the bite of an infected trombiculid mite [1]. This disease can account for up to 23% of all febrile episodes in endemic areas. Mortality rates for scrub typhus range from <1% to 50% depending upon proper antibiotic treatment, status of the individual infected and the strain of *O*. *tsutsugamushi* encountered [2]. The disease is highly endemic in the ‘‘tsutsugamushi triangle” extending from Afghanistan to China, Korea, the islands of the western Pacific and Indian Oceans, and northern Australia [3]. An estimated one billion people are at risk for scrub typhus and approximately one million cases occur annually [4].

The first case of scrub typhus in China was in the city of Guangzhou, in 1948[5]. Before 1986, scrub typhus was mainly distributed in southern provinces and municipalities of China. Since 1990, it has rapidly expanded its region of infection, spreading to the north of Yangtze River [6]. This expansion may be a result of the increasing outdoor activity of urban inhabitants, rapid urbanization of societies, aging populations, and improved public surveillance systems [7]. Jingjiang City, on the north bank of the Yangtze River, had heretofore never reported scrub typhus. If the disease existed in the area previously, its incidence would have been very low and doctors were unaware of its presence. In light of the outbreak of scrub typhus in 2013, we launched an investigation on scrub typhus in Jingjiang City, in order to describe its epidemiological and clinical characteristics, identify risk factors and provide a basis for taking scientific countermeasures.

## Materials and Methods

### Study site

**Fig 1:**
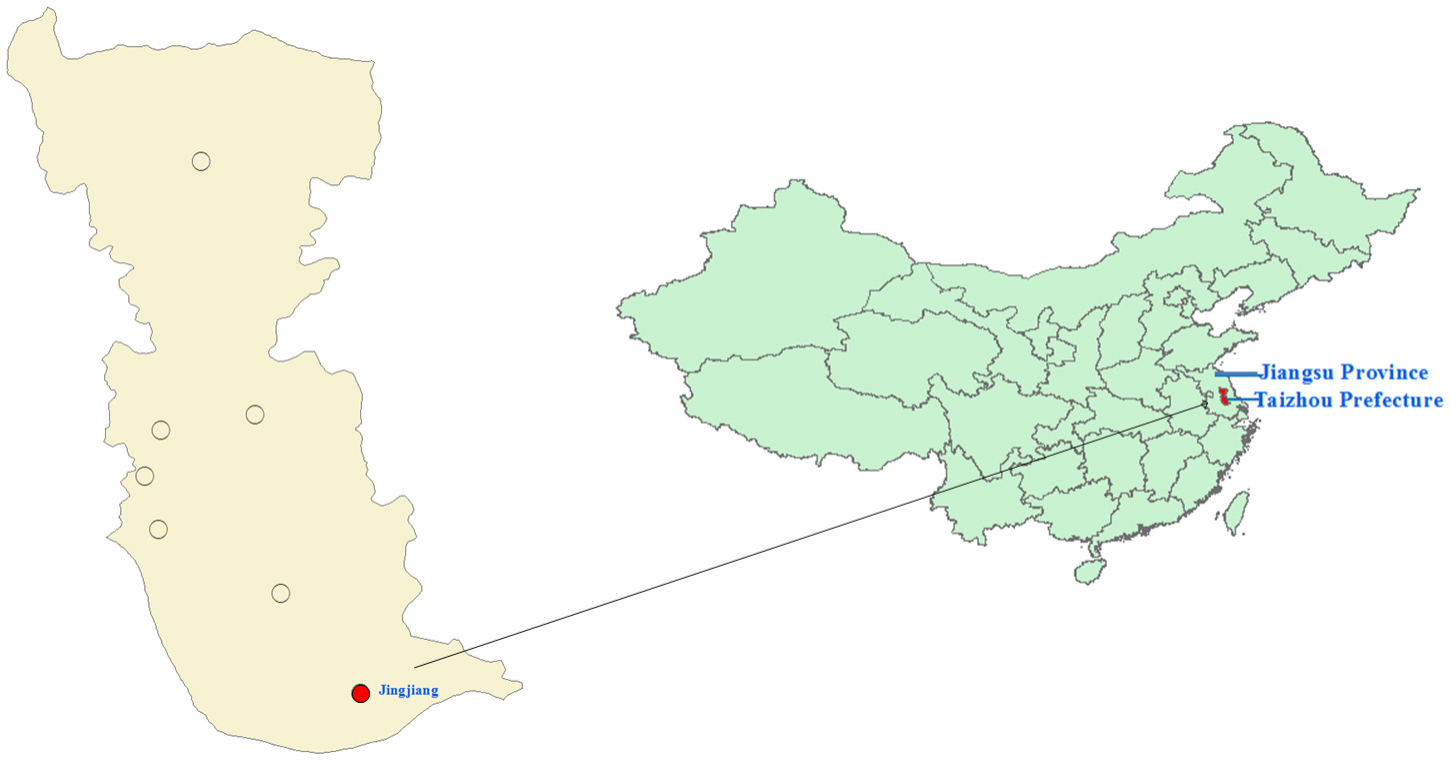
Scrub typhus study site: Jingjiang City, Jiangsu Province, China.

Jingjiang City, with a total population of more than 667,000, is located on the north bank of the Yangtze River in south-central Jiangsu Province, China (longitude, 120°01′~120°33′E; latitude, 31°56′~32°08′N)(Fig 1). The city covers 665km^2^ of alluvial plains with an average altitude of 2.4–4.5m. The area has temperate seasonal weather (average annual temperature: 14.6–15.6°C), adequate rainfall (average annual precipitation: about 1,026 mm, average annual relative humidity: about 74.0%) and adequate sunlight (average annual sunshine duration: about 1908 hours). About 50% of the total population is employed in agricultural production. The main crops farmed include rice, soybean, and sweet potato.

### Case and control definition

A guidebook published by the Chinese Center for Disease Control and Prevention was used as a reference for the diagnosis of scrub typhus [8]. Accordingly, coincidence of three or more of the following items constituted a clinical case of scrub typhus: (1) a field exposure history 1–3 weeks before illness onset; (2) symptoms including high fever, lymphadenopathy, skin rash, splenomegaly, hepatomegaly, or multiorgan dysfunction; (3) typical cutaneous leisions (eschars or ulcers); (4) rapid defervescence with appropriate antibiotics; and (5) Weil-Felix OX-K agglutination titer ≥1:160. Confirmed cases were clinical cases with a positive result (OD>0.5) in immunoglobulinM (IgM) or immunoglobulinG (IgG) by enzyme linked immunosorbent assay (ELISA) [9] or nested polymerase chain reaction (PCR) test targeting a 56-kDa gene of *O*. *tsutsugamushi*[10].

Eligible controls were defined as neighbors of the case subject who were matched for age (within 3 years), sex, and occupation (farmer or non-farmer), local residence time (above 3 months) and who lacked a history of scrub typhus. If an appropriate matched control was not available in the nearest household, then the next nearest household was chosen. A total of 134 cases and 268 controls were recruited from November 2013 through January 2014.

### Data and samples collection

Scrub typhus has been a reportable disease by National Disease Reporting Information System in China since 2006. Every medical institution was required to report scrub typhus cases daily via the web-based surveillance system with unified format, including the information about sex, age, residential address, occupation and date of symptom onset.

A total of 271 outpatient and hospitalized cases were clinically diagnosed with scrub typhus in Jingjiang City from October to December in 2013, according to the criteria described above. However, only 187 patients had complete clinical data and blood samples, and only 134 of these patients agreed to provide information for the investigation of behavioral risk factors of the diseases. Thus, we describe the clinical manifestations of 187 patients with clinical data and 134 patients with behavioral information in this case-controlled study.

Trained interviewers visited the cases and identified matched controls (1:2 pair matching) from cases’ nearest neighbors. A standardized questionnaire was used to collect detailed information on cases and controls. Two main categories of variables were considered in the questionnaire: outdoor activities (work places and type of work engaged in within the 3 weeks prior to the onset of scrub typhus), and potential risky or protective behaviors during outdoor activities.

The study was approved by the Ethics Committee of the Jiangsu Provincial Center for Disease Control and Prevention (JSCDC), the governmental agency in charge of communicable disease control in China. All aspects of the study comply with the Declaration of Helsinki. The Ethics Committee of JSCDC specifically approved the use of verbal informed consent as an alternate to written informed consent, because data were planned to be analysed anonymously. A small amount of blood samples were acquired from human clinical samples, which were collected following medical institutions’ approved procedures and only used for diagnosis of tsutsugamushi disease.

### Laboratory testing

#### Enzyme linked immunosorbent assay (ELISA)

Serum was separated by centrifugation at 2500 g for 10 min and the clots were stored at -70°C pending DNA extraction. All serum samples of patients were tested by IgM and IgG ELISA (InBios International Inc., Seattle, WA, USA). An optical density (OD) >0.5 was considered positive.

#### Detection of the 56-kDa TSA gene

A previously described nested conventional PCR assay targeting the 56-kDa type-specific antigen (TSA) gene was performed [10]. The first PCR product was the template of the second PCR. Reactions were performed in a GeneAmp 9700 thermo cycler (Applied Biosystems, NY, USA). The second amplified products were characterized by electrophoresis of 5 μL of each reaction mixture on a 1.5% agarose gel for 30 min at 100 V.

#### Phylogenetic analyses of the 56-kDa TSA gene

The 56-kDa TSA gene was amplified from the DNA extracted from patients’ blood and cycle sequenced with primers (primer E/primer F and primer C/primer G) [11]. The products were analyzed with an ABI PRISM 3100 Genetic Analyzer (Applied Biosystems, Hitachi, Japan). MEGA 4.0 software[12] was used for the phylogenetic analysis. Homologies among the genotypes were calculated using the Kimura two-parameter model. Phylogenetic trees were assessed using neighbor-joining cluster analysis with 1000 bootstrap replications.

#### Statistical analysis

All statistical analyses were performed by statistical software SPSS version 17.0 (SPSS Inc., Chicago, IL). Risk factors for scrub typhus were identified using univariate and multivariable conditional logistic regression models. Statistical tests were two-sided, with a significance level of 0.05. Univariate analysis of risk factors with significance level of ≤0.05 were included in the multivariable conditional logistic regression model. The backwards stepwise elimination procedure was applied to the multivariable conditional logistic regression model. Consequently, we present odds ratios (ORs) with 95% confidence intervals (CIs) of exposure for various factors.

## Results

### Epidemiological data

**Fig 2:**
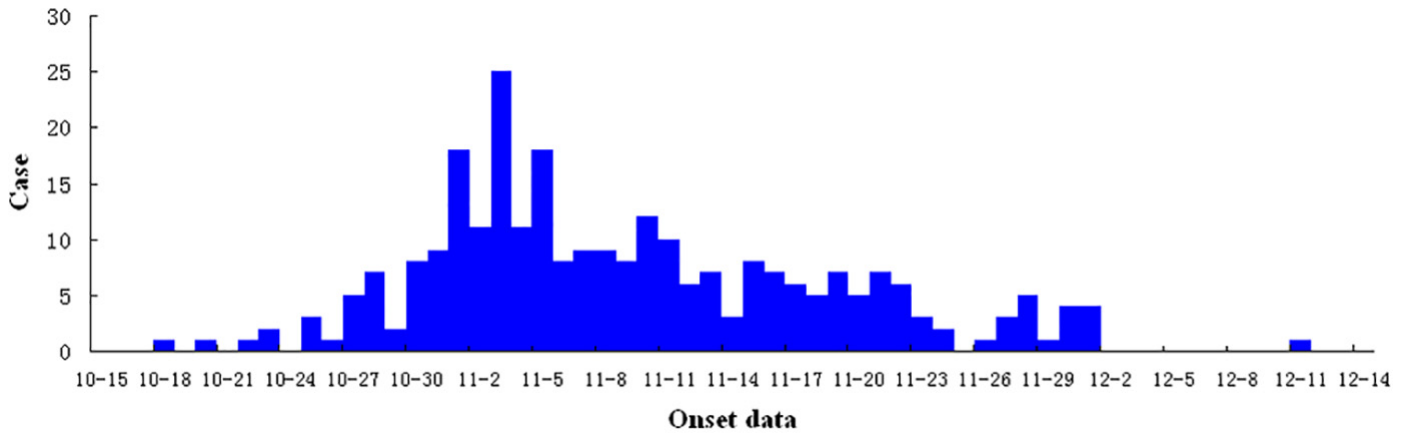
Temporal distribution of onsets of scrub typhus cases in Jingjiang City.

The 271 cases of scrub typhus that occurred from 18 October to 11 December in 2013 were characterized by a peak of onset in early to mid- November (Fig 2), an incidence of 39.6/100,000, and a lack of mortalities following antibiotic treatment. 38.4% of patients were male; and 61.6% were female. Patient ages ranged from 8 to 90 years, with a median age of 59 years. The age distribution was as follows: 0.4% were less than 19 years old; 20.3% were 20 to 49 years old; 59.0% were 50 to 69 years old; and 20.3% were older than 70 years. Patients were predominantly farmers, who accounted for 86.9% of all cases.

### Clinical manifestations and clinical laboratory results

All 187 patients with complete clinical data had high fever and the median of the highest body temperatures was 39.0°C. The duration of fever was 1 to 12 days (average, 4.5 days). Other major clinical manifestations included eschar (88.2%), rash (87.7%), chills (87.7%), headache (66.8%). The proportion of lymphadenopathy was fairly low, only 9.6% (Table 1). The distribution of eschar, which is thought to be the most pathognomonic sign of scrub typhus, was mainly on the chest (22.1%), waist (14.1%), axilla (12.9%), upper limb (11.0%), lower limbs (11.0%), inguinal region (9.2), and neck (8.6%).

**Table 1:** Clinical and laboratory findings for scrub typhus cases of Jinjiang City.

Finding	N*	n**	% of patients
Clinical			
Fever	187	187	100
Eschar or ulcer	187	165	88.2
Rash	187	164	87.7
Chill	187	164	87.7
Headache	187	125	66.8
Conjunctival congestion	187	39	20.9
Lymphadenopathy	187	18	9.6
Bronchopneumonia	187	5	2.7
Laboratory			
Increased WBC count (above 10×10^9^/liter)	140	13	9.3
Decreased platelet count (below 100×10^9^/liter)	137	29	21.2
Elevated transaminase level			
ALT increase	132	78	59.1
AST increase	132	79	59.8
Elevated bilirubin			
Total bilirubin increase	128	13	10.2
Direct bilirubin increase	113	19	16.8
Elevated C-reactive protein	77	70	90.9
Elevated ESR	20	9	45.0

*N: the number of cases with corresponding clinical or laboratory examination

**n: the number of cases with corresponding clinical or laboratory finding

Analysis of clinical laboratory results revealed that only 9.3% of patients had increased white blood cell (WBC) count and 21.2% of patients had decreased platelet (PLT) count. In other words, the majority of patients had normal WBC and PLT count. More than 59.0% of patients had elevated ALT and AST level, but less than 20.0% had elevated total and direct bilirubin level. As a result of inflammation or infection of scrub typhus, 90.9% of patients had elevated C-reactive protein levels and 45.0% had an elevated erythrocyte sedimentation rate (ESR) (Table 1).

### Results of laboratory testing

#### ELISA

A total of 187 serum samples from patients were collected for scrub typhus serological testing. The ELISA results of OD>0.5 were considered positive. 75/187 (40.1%) patients were positive for IgM antibodies, and 19/187 (10.2%) were positive for IgG antibodies, including 3/187 (1.6%) patients’ serum samples with positive IgM and IgG antibodies, respectively (Table 2).

**Table 2:** PCR and ELISA results of 187 cases.

	PCR+ IgM-IgG-	PCR+ IgM+IgG-	PCR- IgM+IgG-	PCR- IgM+IgG+	PCR- IgM-IgG+	Total
**N**	96	6	66	3	16	187

#### Nested PCR

In this investigation, PCR amplification of the 56-kDa TSA gene using DNA extracted from 187 patients’ blood revealed that 102 (54.5%) patients’ samples contained *O*. *tsutsugamushi* DNA, including 6 patients who were IgM positive.

#### Phylogenetic analysis of the 56-kDa TSA gene

**Fig 3:**
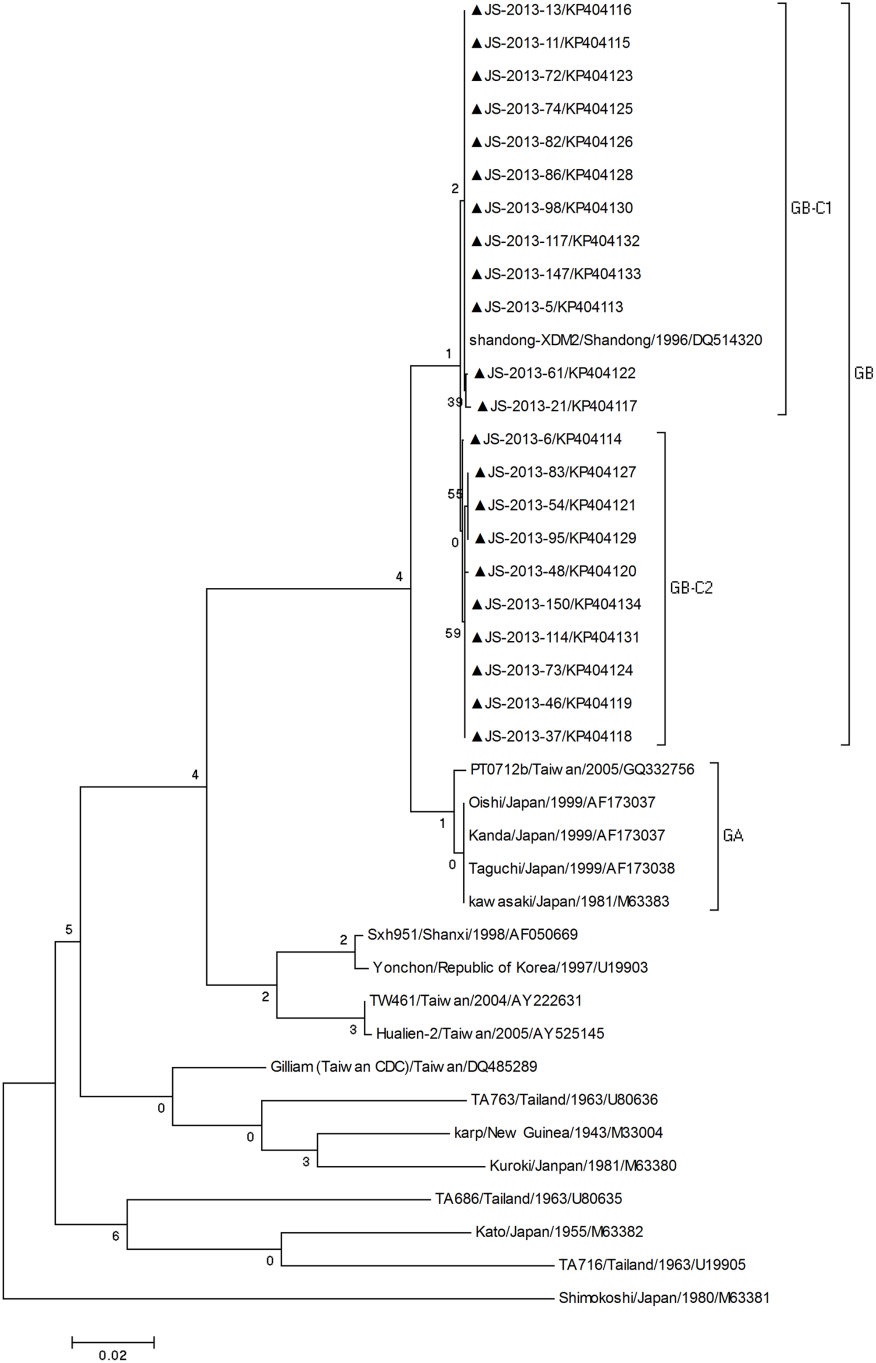
Phylogenetic analysis based on the *O*. *tsutsugamushi* 56-kDa TSA gene. All the sequences of *O*. *tsutsugamushi* in this study marked with black triangles, were compared with other reference sequences available in GenBank. The MEGA 4.0 software was used for the phylogenetic analysis. The stability of the nodes was assessed using neighbor-joining cluster analysis with 1,000 bootstrap replications, and only bootstrap values.70% are shown at the nodes.

Twenty-two independent *O*. *tsutsugamushi* 56-kDa TSA gene sequences (GenBank accession no. KP404113-404134) were acquired from this outbreak. Phylogeny of *O*. *tsutsugamushi* 56-kDa TSA gene sequences detected in this study was determined based on comparisons to reference sequences of *O*. *tsutsugamushi* available through GenBank. The resulting phylogenetic tree showed that the strains in this study clustered with the Kawasaki type group, and displayed sequence similarity to the strain Shandong-XDM2, which was isolated from Shandong Province in 1996 (Fig 3).

### Risk factor analysis by using conditional logistic regression models

On the basis of study requirement, 214 controls and 107 cases were matched by the case-control ratio of 2:1, but for another 27 cases, we were able to find only 27 eligible controls. Eventually, we recruited 134 scrub typhus cases and 241 matched controls. For cases and controls, respectively, average age was 60.2 years and 61.1 years; 35.1% and 36.1% were male; 29.1% and 31.1% had more than 6 years of education; 87.3% and 83.8% were farmers. There were no significant differences in demographic characteristics between cases and controls (Table 3).

**Table 3:** Demographic characteristics of the scrub typhus cases and controls that were enrolled in the study.

characteristic	case(n = 134)	control(n = 241)	*P*
	n(%)or mean±SD	n(%)or mean±SD	
Age	60.2±13.1	61.1±12.1	0.54
Sex			0.84
Male	47(35.1%)	87(36.1%)	
Female	87(64.9%)	154(63.9%)	
Education,years			0.68
≤6 years	95(70.9%)	166(68.9%)	
>6 years	39(29.1%)	75(31.1%)	
Occupation			0.36
Farmer	117(87.3%)	202(83.8%)	
Non-Farmer	17(12.7%)	39(16.2%)	

*SD: standard deviation.

The single-variable conditional logistic regression model revealed that agricultural labor (OR = 2.9, 95%CI 1.5–5.8), working in rice fields (OR = 2.8, 95%CI 1.5–5.0), bundling or moving waste straw (OR = 9.8, 95%CI 5.0–19.2), living at the edge of the village (OR = 0.5, 95%CI 0.4–0.8), and stacking waste straw indoors(OR = 1.6, 95%CI 1.1–2.4) yielded significantly increased scrub typhus. Accordingly, the multiple-variable conditional logistic regression model was constructed using the above risk factors. Risk factors of bundling up or moving waste straw (OR = 9.0, 95%CI 4.6–17.8) and living at the edge of village (OR = 0.6, 95%CI 0.4–0.9) were retained in the final model (Table 4).

**Table 4:** Single- and multiple-variable conditional logistic regression analyses for identifying risk factors for scrub typhus.

Exposure factors or risk behaviors	Missing data	Case	Control	Single-variable regression analysis	Multiple-variable regression analysis
		n(%)	n(%)	OR(95% CI)**	OR(95% CI)**
Agricultural labor	3	125(93.3)	182(76.5)	2.9(1.5–5.8)*	—
Places of agricultural labor					
Rice field	2	122(91.0)	171(71.5)	2.8(1.5–5.0)*	—
Cotton field	8	1(0.8)	1(0.4)	1.4(0.2–10.0)	—
Cornfield	7	2(1.5)	6(2.6)	0.7(0.2–2.8)	—
Soya bean field	1	61(45.9)	128(53.1)	0.8(0.6–1.2)	—
Sweet potato field	3	62(46.6)	120(50.2)	0.9(0.7–1.3)	—
Vegetable field	2	96(72.2)	183(76.0)	0.9(0.6–1.3)	—
Bundling up or moving waste straw	31	115(92.7)	80(36.4)	9.8(5.0–19.2)*	9.0(4.6–17.8)*
Frequency of bundling up or moving waste straw					
none		7(5.6)	139(63.2)	Reference	
1–2 times every two weeks		42(33.9)	59(26.8)	6.9(3.4–14.1)	
3–4 times every two weeks		37(29.8)	19(8.6)	10.9(5.3–22.7)	
≥5 times every two weeks		36(29.0)	2(0.9)	15.7(7.6–32.6)	
Grazing	7	1(0.8)	1(0.4)	1.4(0.2–10.1)	—
Fishing	7	4(3.1)	6(2.5)	1.1(0.4–3.1)	—
Resting on the grass	1	11(8.3)	30(12.4)	0.7(0.4–1.4)	—
Morning exercise outdoor	8	3(2.3)	1(0.4)	2.1(0.7–6.7)	—
Washing by the river	7	34(25.8)	80(33.9)	0.8(0.5–1.1)	—
Living at the edge of villiage	9	34(25.4)	112(48.3)	0.5(0.4–0.8)*	0.6(0.4–0.9)*
Stacking waste straw indoors	10	33(24.6)	29(12.6)	1.6(1.1–2.4)*	—
Living aroud the grass	11	117(88.0)	197(85.3)	1.2(0.7–2.0)	—
The type of fuel					
Gas	10	119(88.8)	196(84.8)	1.3(0.7–2.2)	—
Waste straw	12	99(73.9)	158(69.0)	1.2(0.8–1.7)	—
Firewood	15	59(44.4)	130(57.3)	0.7(0.5–1.0)	—
Raising poultry and livestock	10	61(45.5)	99(42.9)	1.1(0.8–1.5)	—
Mouse activities indoors	0	92(68.7)	131(54.4)	1.4(0.9–2.2)	—
Similar cases in the family	0	2(1.5)	3(1.2)	1.1(0.3–4.4)	—

*:*P*≤0.05.

**:OR = odds ration;CI = confidence interval.

Finally, we analyzed the correlation between the frequency of bundling or moving waste straw, which was expressed with a dummy variable, and contracting scrub typhus. Compared to not bundling or moving waste straw, odds ratios of 1–2 times, 3–4 times and ≥5 times every two weeks was 6.9, 10.9 and 15.7, respectively.

## Discussion

The epidemiological data of scrub typhus outbreak in Jingjiang City showed that the majority of patients were female farmers 50 to 69-years-old, which might be explained by the fact that most young, male individuals of rural families work in urban areas to alleviate their economic burden, and that the elderly and women are left to do farm work. The disease occurred during the fall harvest season, and 1–2 weeks before the outbreak there had been moderate rainfall in the area. The epidemic patterns of scrub typhus in China include three types: 1. summer scrub typhus endemicity south of the Yangtze River; 2. autumn-winter scrub typhus endemicity north of the Yangtze River; and 3. northern China endemicity where there is evidence of *O*. *tsutsugamushi* in animals but no patients reported to be diagnosed with the disease[13]. Discovery of scrub typhus in Jingjiang City falls into the second epidemic pattern. Meanwhile, a vector survey in November 2013 demonstrated that *Leptotrombidium scutellare* was the dominant species of mites in Jingjiang City, which conforms to the results of previous studies[5,13].

Fever was the most common clinical symptoms in the present case series (100% cases) in contrast to other reports from India where headache and myalgia (93.8%) were the most common manifestations of scrub typhus followed by fever (71.9%)[14]. Unlike reports from other studies, the clinical presentations in this study’s cases were peculiar in the sense that 88.2% of cases presented with eschar or ulcer, and 87.7% presented with rash. In these cases, the eschar or ulcer, which results from a painless chigger bite, is the pathognomonic sign of scrub typhus and was located most commonly in areas that are relatively easy to examine, including chest (22.1%), waist (14.1%), upper limb (11.0%), lower limbs (11.0%). Lymphadenopathy and leucocytosis, which are features of the disease, were not prominent in the present study. This pattern of symptoms was similar to results of other studies of scrub typhus from the north of China [15,16].

The phylogenetic analysis showed that the Kawasaki type strains of *O*. *tsutsugamushi* were the major genotype in these areas and widely distributed throughout Jiangsu Province[17]. The phylogenetic tree showed two groups within the Kawasaki type group: Group A and Group B. Group A included the Japanese isolates Kawasaki, Kanda, Oishi and Taguchi and Taiwan isolate PTO712b. In this study, our strains all belonged to Group B and were differentiated as two clusters (GB-C1 and GB-C2). C1 strains showed similarity to the strain Shandong-XDM2 isolated from Shandong Province. C2 strains had unique sequences in GenBank. In conclusion, our study suggests that the new cluster (GB-C2) and the previously reported cluster (GB-C1) of *O*. *tsutsugamushi* were associated with scrub typhus outbreak in Jingjiang City.

Based on conditional logistic regression analysis, bundling or moving waste straw was associated with a significantly higher risk of contracting scrub typhus. Despite its impact on air quality, burning on the farm fields has been widely adopted by Chinese farmers as an easy and cheap way to remove stalks after harvests. However, many people are increasingly concerned with the environmental consequences of China’s rapid economic development. Thus, in order to preserve air quality, local farmers in Jingjiang City no longer burn waste straw in the farmland and, instead, bundle waste straw and move it to the footpaths of the villages. Eventually, this waste straw is picked up and recycled by straw-recycling companies. In harvest season, the waste straw soaked with rainwater provides good habitats for chiggers. Thus bundling or moving waste straw, which increases contact between humans and chiggers, became the major risk factor for scrub typhus infection. The waste straws were stacked alongside the footpaths which were located inside the village, so those living at the edge of village had a lower chance of contacting the waste straw and lower risk of infection by scrub typhus compared to those living in the center of village.

Our study has some limitations. Firstly, the surveillance data were obtained from a passive reporting system, which means that some cases of scrub typhus might have gone unreported. Secondly, genotyping was conducted by nested PCR and sequencing using DNA extracted from case specimens, but we failed to isolate *O*. *tsutsugamushi* from patients’ blood by suckling mice inoculation and cell culture (Vero and L929). Finally, our vectors were not all properly certified. 45 mites of a vector survey were recorded only *Leptotrombidium scutellare* in November 2013, including 18 mites collected from the body surface of 2 Suncus murinus outdoors, and 27 mites collected from agricultural fields. Unfortunately, *O*.*tsutsugamushi* was unable to be detected from vectors.

Despite these limitations, our study presents valuable results using a community-based case-control study that can contribute to the establishment of an evidence-based intervention strategy to reduce the incidence of scrub typhus. The behavioral changes of waste straw disposal has directly induced scrub typhus outbreak in Jingjiang City. Based on our results, one simple and practical measure for the prevention of this disease would be to educate the community, to avoid direct contact with waste straw, and use automated machines to bundle or move waste straw, then transport waste straw from villages. Meanwhile, individuals frequently engaging in agricultural labor should be advised to apply available insect repellant on body parts to prevent mite bites while outdoors. Finally, it is necessary to carry out further strategies such as the implementation of rodent and vector control, strengthen surveillance and a training program for medical staff in order to minimize future epidemics.
